# Supplementary material for: DMRforPairs: identifying Differentially Methylated Regions between unique samples using array based methylation profiles
Source: BMC Bioinformatics. 2014 May 15;15:141. doi: 10.1186/1471-2105-15-141 (PMC4046028; doi:10.1186/1471-2105-15-141)
Supplement: Additional file 2 — DMRforPairs output for the comparison of A431-MCF7 and NA17018-NA17105. Please start from the HTML files in each folder. Available via the BMC Bioinformatics website. [file 1471-2105-15-141-S2.zip › 1394847754114233_MOESM2_ESM/A431_MCF7/figures/26607.pdf]

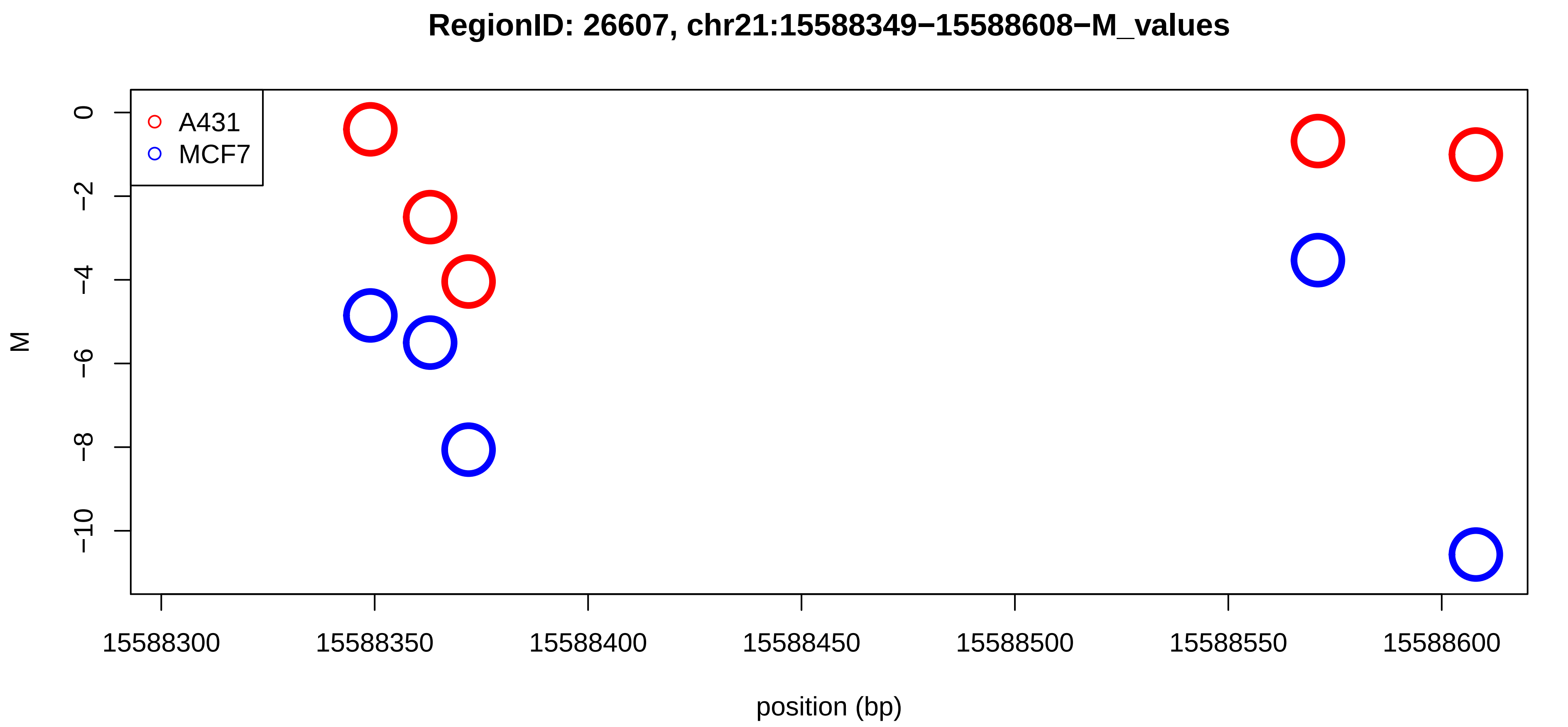
RegionID: 26607, chr21:15588349−15588608−M_values
A431
MCF7
M
0
−2
−4
−6
−8
−10
15588300
15588350
15588400
15588450
15588500
15588550
15588600
position (bp)

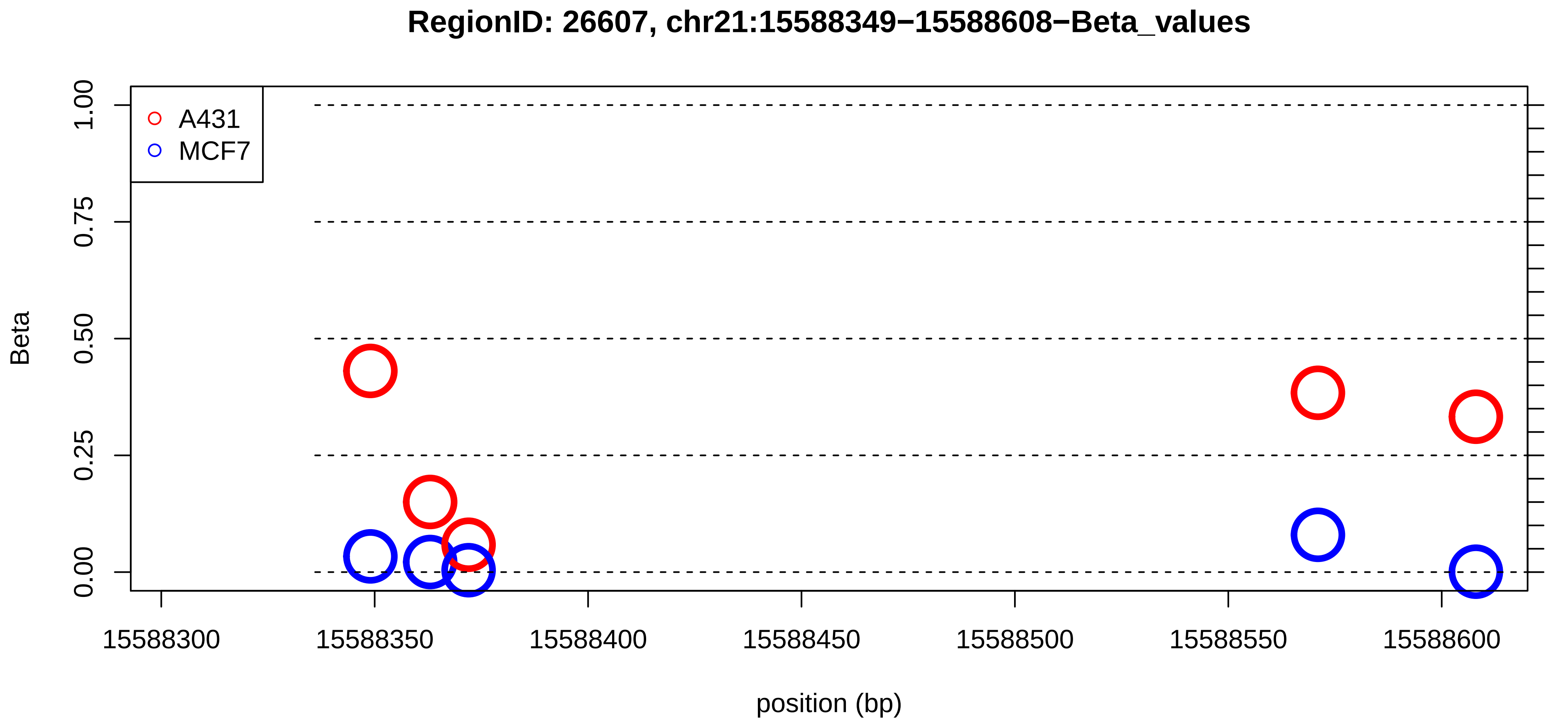
RegionID: 26607, chr21:15588349−15588608−Beta_values
A431
MCF7
Beta
1.00
0.75
0.50
0.25
0.00
15588300
15588350
15588400
15588450
15588500
15588550
15588600
position (bp)
